# Internet-based behavioural activation therapy versus online psychoeducation for self-reported suicidal ideation in individuals with depression in Indonesia: a secondary analysis of an RCT

**DOI:** 10.1136/bmjment-2023-300918

**Published:** 2024-02-20

**Authors:** Caroline B B C M Heuschen, Koen Bolhuis, Jasper B Zantvoord, Retha Arjadi, Damiaan A J P Denys, Maaike H Nauta, Anja Lok, Claudi L Bockting

**Affiliations:** 1 Department of Psychiatry, Amsterdam UMC Location AMC, Amsterdam, The Netherlands; 2 Department of Child and Adolescent Psychiatry/Psychology, Erasmus MC Sophia Children's Hospital, Rotterdam, The Netherlands; 3 Amsterdam Neuroscience, Amsterdam, The Netherlands; 4 Faculty of Psychology, Atma Jaya Catholic University of Indonesia, Jakarta, Indonesia; 5 Department of Clinical Psychology and Experimental Psychopathology, University of Groningen, Groningen, The Netherlands; 6 Amsterdam Public Health Research Institute, Amsterdam UMC, University of Amsterdam, Amsterdam, The Netherlands; 7 Centre for Urban Mental Health, University of Amsterdam, Amsterdam, The Netherlands

**Keywords:** suicide & self-harm, depression & mood disorders, depression

## Abstract

**Background:**

Southeast Asia has the highest suicide mortality worldwide. To improve our knowledge on the effectiveness of interventions for suicidal ideation (SI) in individuals with depression in Indonesia, we conducted a secondary analysis of a randomised controlled trial.

**Objective:**

We explored whether an internet-based behavioural activation (BA) intervention (‘Guided Act and Feel Indonesia’ (GAF-ID)) was superior in targeting SI compared with online-delivered psychoeducation (PE).

**Methods:**

In total, 313 participants were randomised between treatment allocation. The SI item of the Patient Health Questionnaire-9 was the primary outcome measure. Mediation analyses were conducted to identify if BA at week 10 mediated the relationship between intervention and SI at week 24.

**Findings:**

The GAF-ID intervention was not superior in reducing SI compared with online minimal PE at week 10 (OR 0.61, 95% CI (0.37 to 1.01)), nor at week 24 (OR 0.84, 95% CI (0.47 to 1.52)). SI at week 24 was not mediated by BA at week 10 (b=−0.03, 95% CI (−0.05 to 0.00), p=0.07).

**Conclusions:**

In individuals with depression in Indonesia, the GAF-ID intervention was not superior in reducing self-reported SI compared with PE. Also, the association between treatment condition and SI at week 24 was not mediated via BA at week 10.

**Clinical implications:**

This study supports the need for further research on the efficacy of psychological treatments targeting SI in the Southeast Asia context.

**WHAT IS ALREADY KNOWN ON THIS TOPIC**

- Effective interventions for suicidal ideation (SI) in individuals with depression remain scarce.

**WHAT THIS STUDY ADDS**

- We showed that the effect of Guided Act and Feel Indonesia on SI at week 24 was not mediated via behavioural activation at week 10.

**HOW THIS STUDY MIGHT AFFECT RESEARCH, PRACTICE OR POLICY**

- This study supports the need for further research on the efficacy of psychological treatments for SI in Southeast Asia.

## Introduction

Southeast Asia has the highest suicide mortality worldwide, accounting for 39% of all suicides globally.^1 2^ Although suicide mortality is estimated at a relatively low rate of 2.6 per 100 000 in Indonesia,^1^ a recent analysis suggests that Indonesia has the highest number of unreported suicides in the world.^3 4^ Suicidal ideation (SI) is a strong predictor of increasing severity of suicidal behaviour (SB) and is, therefore, an important target for suicide prevention.^5^ Approximately 33% of individuals with depression experience SI.^6^ In Indonesia, depression is ranked as one of the leading causes of disability-adjusted life years (ref). Currently, there are still unmet needs in the assessment and treatment of individuals with depression and SI.^7^ Moreover, studies identified specific cultural and social risk factors for suicide in Southeast Asia, such as the level of religious involvement and severity of interpersonal problems.^8^ Also, compared with high-income countries (HICs), the male-to-female ratio for suicide is closer to 1,^2^ and the overall reported prevalence of mental health conditions is lower in Southeast Asia.^2 9^

A situation analysis in Indonesia identified a lack of high-quality research on suicide, which has contributed to the implementation of suicide prevention efforts that are not data-driven, and employment of strategies developed in HICs, despite numerous cultural and demographic differences.^4^ In addition, access to mental healthcare remains severely limited with only 3 psychiatrists and 1.7 psychologists per 1 million inhabitants.^1^ However, with increasing internet access across the globe, online interventions are a promising avenue to narrow the gap in mental healthcare services.^10^ Moreover, recent studies have shown that digital psychological interventions are effective in individuals with depression in low-income and middle-income countries (LMICs).^11^ In addition, online interventions can lower the threshold to seek help, which makes them especially suitable for individuals with SI.^5^

Given the importance of decreasing SI in individuals with depression, we conducted a secondary analysis of a randomised controlled trial (RCT) in Indonesia.^11^ This RCT (n=313) showed that guided online behavioural activation efficaciously reduced depressive symptoms and increased remission rates of depression as compared with online minimal psychoeducation (PE) in Indonesia.^11^ Behavioural activation (BA) focuses on the behaviour that occurs jointly with ruminative thoughts or SI—as opposed to the thought content per se.^12^ BA leads to increased pleasant activities or problem-solving strategies, resulting in improved mood.^12^ Studies have also suggested that BA can lead to reduced SI via increased activity.^13^

The aim of the current study was twofold, first to study whether the internet-based BA intervention, supported by lay counsellors, called ‘Guided Act and Feel Indonesia’ (GAF-ID) was superior to online minimal PE in reducing SI in individuals with depression in Indonesia. Second, we conducted a temporally ordered mediation analysis to explore the extent to which the association between treatment condition and SI at week 24 was mediated via BA at week 10. Both the direct and indirect effects of treatment groups were estimated. In line with our primary hypothesis, we studied BA as a potential mediator as it is through BA that the GAF-ID intervention exerts its primary effect. In addition, as a secondary analysis, we assessed the extent to which the association between treatment condition and SI at week 24 was mediated by (other) depressive symptoms at week 10.

## Methods

### Study design

This study is a secondary analysis of a two-group RCT of an internet-based BA programme in individuals with major depressive disorder (MDD) (n=313).^14^

### Interventions

The intervention group received the internet-based BA intervention (GAF-ID).^11 14^ The GAF-ID consists of a series of eight structured modules that can be completed over 8 weeks.^11 14^ Participants in the control group received online-delivered minimal PE without additional support.^14^ Details of the original RCT are reported elsewhere.^11 14^

### Participants and randomisation

In total, 313 participants were included and randomised into the treatment (n=159) vs control (n=154) groups.^11^ Eligible participants were aged 16 years or older, scored ≥10 on the Patient Health Questionnaire-9 (PHQ-9), had a diagnosis of MDD or persistent depressive disorder assessed by Diagnostic and Statistical Manual of Mental Disorders, Fifth Edition or Structured Clinical Interview for DSM-5 (SCID-5), were proficient in the Indonesian language Bahasa and had fluency in using the internet.^14 15^ Participants with current substance use disorder, a current or previous manic or hypomanic episode, a psychotic disorder or acute suicidality defined as a suicide plan with preparatory behaviour, assessed with the SCID-5- Research Version^11^ were excluded, as were those receiving psychological interventions for mental health complaints.^14^ In total, only two participants (1.5%) were excluded due to acute suicidality.^11^ Participants were recruited via online self-referral, and referral from mental health institutions or mental health professionals.^14^

### Assessments

Sociodemographic information was collected at baseline. In addition, the PHQ-9,^16^ the Indonesian version of the Inventory of Depressive Symptomatology Self-Report (IDS-SR)^17^ and the Behavioural Activation for Depression Scale-Short Form (BADS-SF) were completed every 2 weeks.^18^ Following the protocol paper of the original RCT,^14^ the PHQ-9 was used as the primary outcome measure. The PHQ-9 is a self-reported questionnaire to assess depressive symptoms and is the most frequently used screener for depression globally. The PHQ-9 has been validated for the Indonesian population.^16 19^ The IDS-SR was used as a secondary outcome measure. The IDS-SR is a self-reported depression-symptom scale to assess severity of depressive symptoms.^17^

### Assessment of self-reported SI

We assessed SI with item 9 of the PHQ-9 (“Over the past 2 weeks, how often have you been bothered by thoughts that you would be better off dead or of hurting yourself in some way?”).^16^ For sensitivity analyses, SI was assessed by the Indonesian version of the IDS-SR item 18 (‘Thoughts of death or suicide’).^17^ The approach of assessing SI using a single item from a depression scale is in line with earlier studies.^6 20^ For all analyses, SI was recoded as a dichotomous variable due to lower numbers on higher scores of the items (online supplemental file, page 1). To this end, we created two categories: no SI (score of 0) vs the presence of SI (scores ≥1). This cut-off value has been commonly used in previous studies.^21–24^

10.1136/bmjment-2023-300918.supp1 Supplementary data

### Assessment of self-reported depressive symptoms

Depressive symptoms were assessed using the PHQ-9. For sensitivity analyses, the Indonesian version of the IDS-SR was used.^17^ The PHQ-9 and IDS-SR were measured at baseline, week 10 and week 24.^14^ We created depression sum scores of PHQ-9 and the IDS-SR with the exclusion of SI items (PHQ-9 item 9, IDS-SR item 18) from the total score to test SI scores independently from the sum scores of depressive symptoms.

### Assessment of self-reported behavioural activation

We assessed BA with the BADS-SF.^18^ The BADS-SF is a self-reported 9-item questionnaire that measures changes in activation and avoidance in the previous week. A higher score corresponds to an increase in BA. The validity and reliability of BADS-SF have been established in previous work.^18^ The BADS-SF was measured at baseline, week 10 and week 24.

All assessments were administered at baseline and every 2 weeks thereafter up to the main post-treatment evaluation at week 10 (end point), with follow-up at 12 and 24 weeks after baseline. For the purposes of the current study, depressive symptoms, BA levels and SI were studied at baseline, week 10 and week 24 (table 1).

**Table 1:** Assessments

Measures	Description	Baseline (t0)	Week 10	Week 24
Potential mediators				
BADS-SF	Behavioural activation		+	
SUM PHQ-9	Sum score of depressive symptoms		+	
SUM IDS	Sum score of depressive symptoms		+	
Primary outcome				
PHQ-9 item 9	Suicidal ideation			+
Secondary outcome				
IDS-SR item 18	Suicidal ideation			+
Demographics	Sociodemographic characteristics	+		
Clinical information	Information related to clinical conditions	+		

The time frames of 10 weeks and 24 weeks are counted from baseline (applied in both groups).

BADS-SF, Behavioural Activation for Depression Scale Short Form; IDS-SR, Inventory of Depressive Symptomatology Self-Report; IDS-SR item 18, ‘Thoughts of death or suicide’; PHQ-9, Patient Health Questionnaire-9; PHQ-9 item 9, "Over the past 2 weeks, how often have you been bothered by thoughts that you would be better off dead or of hurting yourself in some way?"; SUM PHQ-9 and SUM IDS, sum scores of PHQ-9 and the Indonesian IDS-SR were created while excluding the item related to suicidal ideation (PHQ-9 item 9, IDS-SR item 18, respectively) from the total score; 10 weeks and 24 weeks are counted from baseline (applied in both groups).

### Data analysis

The association between treatment allocation and SI measured at baseline, week 10 and week 24, respectively, was calculated in ORs. The 95% CI was used to estimate the precision of the OR at each time point. A simplex mediation model was adopted to explore the extent to which the association between treatment condition and SI at week 24 was mediated via BA at week 10. Mediation analysis estimates the direct and indirect effects of the exposure on an outcome and is especially useful for identifying important mediating factors that can explain treatment effects.^25^ In line with the literature stating that correlation is neither a necessary nor a sufficient condition of causality, we conducted mediation analyses independent of whether the total effect path, the association between exposure and outcome, was statistically significant.^25^ Our main interest was whether the association between treatment condition and SI at week 24 was mediated via BA at week 10. In addition, we explored whether the association between treatment condition and SI at week 24 was mediated via depressive symptoms at week 10.

**Figure 1:**
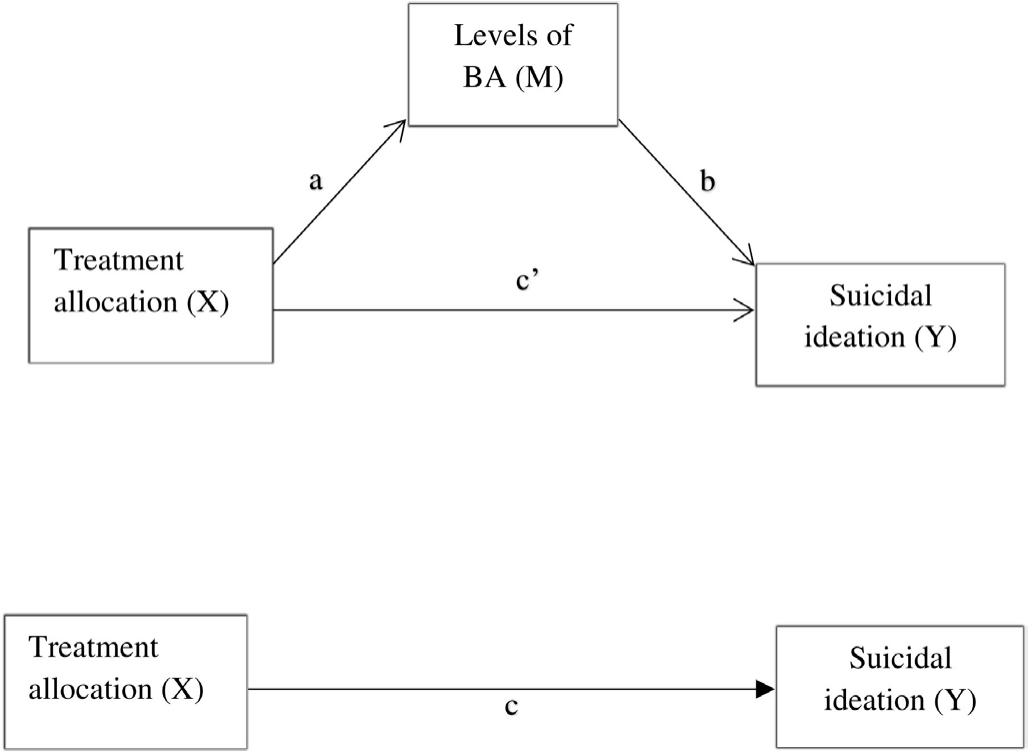
Simplex mediation model.

We refer to the path estimating the relationship between exposure (treatment condition) and BA (mediator) as the a path, and refer to the path between BA and SI (outcome) as the b path. The direct effect of the treatment condition on SI is noted as the c path, and after accounting for the mediator (ie, BA) as c*’* path. A visualisation of the adopted mediation model is illustrated in figure 1. The PE group was used as a control group. To enable mediation analysis, a linear regression was conducted to estimate path a, and a binomial logistic regression was performed to estimate paths b and c*’*. The product of a*×*b coefficients method was used to indicate the indirect effect. The CIs of a*×*b were bootstrapped 1000 times. A mediated effect was considered statistically significant if the 95% CI of the indirect effect coefficient did not include zero. No covariates were included in the analyses as no baseline differences were observed between the two randomised treatment groups. Moreover, the inclusion of potential confounders age, gender, marital status, socioeconomic status, education level and occupation did not change the regression coefficient for BADS-SF as potential mediator (path b) at week 10. The *mediation* R package was used to conduct the mediation analyses.^26^

Missing values on BADS-SF assessment at weeks 2, 4, 6, 8 and 10, PHQ-9 item 9 at week 10 and week 24, IDS-SR item 18 at week 10 and week 24, SUM PHQ-9 at week 10 and SUM IDS at week 10 were imputed by multiple imputation, under the MAR assumption, using the *mice* R package.^27 28^ In total, 14 imputations were used.^28^ To impute missing values, baseline variables, treatment allocation and available repeated measurements were used.

We conducted sensitivity analyses using the Indonesian version of the IDS-SR. The sensitivity analyses were identical in methodology to the former analyses. Considering that we studied four pathways which could have possibly led to multiple testing, a Bonferroni correction of α=0.01 was applied. All statistical analyses were performed using R V.3.6.3 and two-tailed p values <0.01 were taken as a cut-off for statistical significance.

## Results

**Figure 2:**
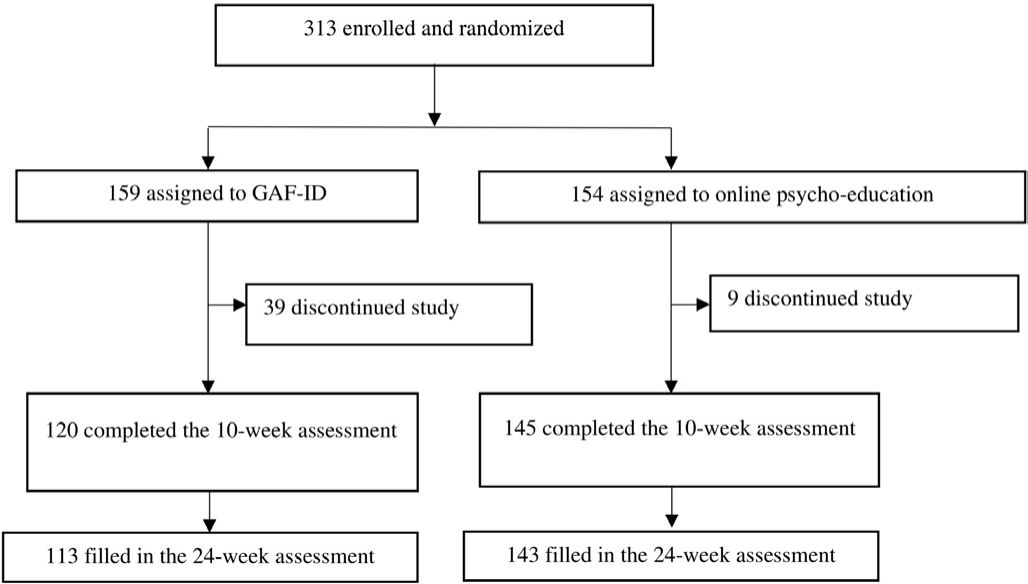
Flow chart. GAF-ID, Guided Act and Feel Indonesia.

Descriptive information on participants at baseline is presented in table 2. Median age of the study population was 24 years (range 16–51) and 82% of participants were female. The baseline characteristics were comparable across intervention groups, indicating successful randomisation (table 2). In total, 313 participants with MDD were enrolled, and 159 participants were randomised to the treatment group (GAF-ID) and 154 to the control group (PE). At week 10, 39 participants (25%) had discontinued the study in the GAF-ID group and 9 participants (6%) discontinued in the control group (figure 2). The frequency of dropouts was significantly higher in the GAF-ID group (OR 0.19, 95% CI (0.09 to 0.41), p<0.001).^11^ Participants who dropped out had significantly lower depression scores at baseline than those who remained in the study, but there were no statistical differences in age or gender.^11^ At week 24, an additional seven participants (6%) discontinued the study in the GAF-ID group and two participants (1.4%) in the control group (figure 2).

**Table 2:** Characteristics at baseline (n=313)

		Treatment group (GAF-ID)	Control group (PE)	Between-group statistics				
		(n=159)	(n=154)	χ^2^	t-test	Mann-Whitney U test	df	P value
Female	N (%)	128 (81%)	125 (81%)	0.02			1	0.88
Age	Mean (SD)	24.5 (4.9)	24.5 (5.2)		−0.13		311	0.90
Education level	Levels	3/61/6/76/13	2/59/12/73/8	3.41			4	0.49
SES level	Levels	32/98/29	27/100/27	0.44			2	0.80
Occupation	Levels	18/3/56/6/4/13/57/2	6/7/48/3/4/17/63/6	11.97			7	0.10
Marital status	N (%)	141 (89%)/17 (11%)/1 (0.6%)	135 (88%)/16 (10%)/2 (1%)	1.42			3	0.70
Mental Illness self below 16th	N (%)	23 (15%)	19 (12%)	0.31			1	0.58
Mental Illness self above 16th	N (%)	45 (28%)	44 (29%)	0.00			1	0.96
Previous depressive episodes (n)	Levels	76/59/18/3/2/1	75/57/17/4/1	1.47			5	0.92
Suicide attempt self below 16th	N (%)	24 (15%)	14 (9%)	2.64			1	0.10
Suicide attempt self above 16th	N (%)	28 (18%)	22 (14%)	0.64			1	0.42
Suicide attempt parent below 16th	N (%)	4 (3%)	5 (3%)	0.15			1	0.70
Suicide attempt parent above 16th	N (%)	1 (0.6%)	5 (3%)	2.85			1	0.09
Thoughts that you would be better off death	Levels	60/47/20/32	61/44/18/31	0.15			3	0.99
Thoughts about my own death or suicide	Levels	45/75/25/14	49/74/28/3	7.39			3	0.06
PHQ-9 sum (suicide item excluded)	Mean rank	156.1	157.9			12112.5		0.87
IDS-SR sum (suicide item excluded)	Mean (SD)	40.1 (10.8)	41.1 (9.3)		- 0.10		311	0.92
BADS-SF sum	Mean rank	159.1	154.8			11907.5		0.68
PTSD (SCID-5)	N (%)	22 (14%)	30 (20%)	1.80			1	0.18

Education levels: junior high school/senior high school/vocational/bachelor/master.

SES level (from monthly expenses): low (<1 000 000 IDR)/middle (between 1 000 000 and 5 000 000 IDR)/high (>5 000 000 IDR).

Occupation: unemployed/professional/private sector/civil employee/entrepreneur/freelancer/college student/housewife.

Marital status: not married yet/married/divorced. Previous depressive episodes: 0/1/2/3/4/5.

PHQ-9 item 9: thoughts that you would be better off dead: 0/1/2/; IDS-SR item 18: thoughts of my own death or suicide: 0/1/2/3.

BADS-SF, Behavioural Activation for Depression Scale-Short Form; GAF-ID, Guided Act and Feel Indonesia; IDS-SR, Indonesian version of the Inventory of Depressive Symptomatology Self-Report; PE, psychoeducation; PHQ-9, Patient Health Questionnaire-9; PTSD, post-traumatic stress disorder; SCID-5, Structured Clinical Interview for DSM-5; SES, socio-economic status.

### Primary aim: comparison of interventions main effect on SI

**Figure 3:**
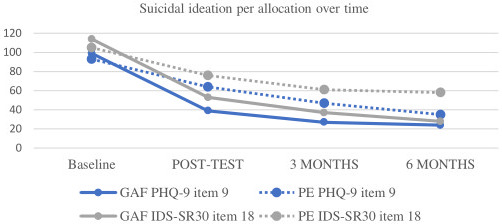
Suicidal ideation scores PHQ-9 vs IDS-SR. GAF, Guided Act and Feel; IDS-SR, Indonesian version of the Inventory of Depressive Symptomatology Self-Report; PE, psychoeducation; PHQ-9, Patient Health Questionnaire-9.

We did not find a significant difference in SI assessed by PHQ-9, between the GAF-ID and PE group at week 10 (OR=0.61, 95% CI (0.37 to 1.01), p=0.05) nor at week 24 (OR 0.84, 95% CI (0.47 to 1.52), p=0.56) (table 3). However, we did find that participants in both groups reported reduced SI over time (figure 3); a decrease of 29% in the GAF-ID group and 16% in the PE group from baseline to week 10 (figure 3), and a 41% decrease in the GAF-ID group and a 36% decrease in the PE group from baseline to week 24 (figure 3). Sensitivity analysis with the IDS-SR showed similar findings at week 10 (OR 0.72, 95% CI (0.44 to 1.17), p=0.18), although at week 24 there was a significant difference in SI between treatment groups, favouring the GAF-ID group (OR 0.49, 95% CI (0.28 to 0.84), p<0.01) (online supplemental table 1). A post hoc cross-sectional analysis at week 10 showed that individuals with SI reported lower BA levels compared with individuals without SI in both the GAF-ID and PE groups (online supplemental figure 1).

**Table 3:** Suicidal ideation

PHQ-9 item 9	Treatment group (GAF-ID)	Control group (PE)	OR	95% CI	P value
At baseline	99 (62%)	93 (60%)	1.08	0.69 to 1.71	0.73
Week 10	39 (33%)	64 (44%)	0.61	0.37 to 1.01	0.05
Week 24	24 (21%)	35 (24%)	0.84	0.47 to 1.52	0.56

PHQ-9 item 9: thoughts that you would be better off dead (0/1/2/3) recoded into two categories: no suicidal ideation (score of 0) vs the presence of suicidal ideation (scores ≥1).

GAF-ID, Guided Act and Feel Indonesia; PE, psychoeducation; PHQ-9, Patient Health Questionnaire-9.

### Secondary aim: mediation analyses

#### Primary analysis

The mediation analysis showed no mediation effect via BA at week 10 on SI (measured with PHQ-9) at week 24 (b=−0.03, 95% CI (−0.05 to 0.00), p=0.07) (table 4). Sensitivity analysis showed preliminary support for mediation via BA at week 10 on SI (measured with IDS-SR) at week 24 in favour of the GAF-ID group (b*=*−0.03, 95% CI (−0.06 to –0.00), p<0.03) (online supplemental table 2), meeting the criterium for temporal ordering and indicating total mediation. However, after the Bonferroni correction for multiple testing, the mediation effect was no longer statistically significant (p>0.01).

**Table 4:** Temporally ordered mediation models (n=313)

Mediator week 10	Outcome week 24	Direct effect				Indirect effect			
		Estimate	SE	95% CI LL to UL	P value	Estimate	SE	95% CI LL to UL	P value
BADS-SF	PHQ-9 item 9	−0.00	0.05	−0.10 to 0.10	0.96	−0.03	0.01	−0.05 to 0.00	0.07
SUM PHQ-9	PHQ-9 item 9	0.01	0.05	−0.08 to 0.11	0.82	−0.04	0.02	−0.07 to –0.01	0.01*

Direct effect: path c’; indirect effect: shown as indirect effect coefficient (function of the compound pathway ab). SUM PHQ-9; the sum score of PHQ-9 was created while excluding the item related to suicidal ideation (PHQ-9 item 9) from the total score.

BADS-SF, Behavioural Activation for Depression Scale-Short Form; LL, lower limit; PHQ-9, Patient Health Questionnaire-9; UL, upper limit.

Post hoc analyses with BA measured with the BADS-SF at weeks 2, 4, 6 and 8, respectively, as mediator, and with SI measured with PHQ-9 at week 10 as outcome showed preliminary support for mediation via BA at weeks 6,and 8, respectively, on SI at week 10 (online supplemental table 3).

#### Secondary analysis

Mediation analysis showed support for mediation via depressive symptoms at week 10 on SI (measured with PHQ-9) at week 24 (b=−0.04, 95% CI (−0.07 to –0.01), p<0.01) (table 4), meeting the criterium for temporal ordering. Sensitivity analysis showed support for mediation via depressive symptoms at week 10 on SI (measured with IDS-SR) at week 24 (online supplemental table 2) but, again, this mediation effect was no longer significant after Bonferroni correction for multiple testing (p>0.01).

## Discussion

Our study showed that the GAF-ID intervention was not superior in reducing SI compared with online minimal PE both at week 10 and week 24 in individuals with MDD in Indonesia. In addition, in contrast to our primary hypothesis, the association between treatment condition and SI at week 24 was not mediated via BA at week 10. We did find support for mediation via depressive symptoms at week 10 on SI at week 24, in favour of the GAF-ID group, in line with our secondary hypothesis.

Our findings illustrate no superiority in alleviation of SI with the GAF-ID intervention compared with online minimal PE. One possible explanation for the absence of superiority of GAF-ID relative to minimal PE could be related to the decrease of SI over time in the PE group, which might in turn be associated with the occurrence of BA in the PE group. Another possibility for not finding a difference between treatment groups, could be the exclusion of patients with acute suicidality, thereby excluding patients who could potentially benefit most from treatment.^11^ Moreover, the assessment of SI, using a single item from self-reported questionnaires, could have influenced the measurement of SI.

Current evidence of the effectiveness of psychological treatments for SI in the context of depression is inconsistent. Several studies have shown no effect of short-term psychotherapy or cognitive behavioural therapy (CBT) on SI,^29^ whereas other studies showed positive effects on SI when interpersonal therapy, a form of (online) CBT, or a combination of cognitive or problem-solving approaches with a BA intervention or a short-term behavioural intervention was provided.^5 6 13 30^ Together with our findings, these studies affirm the ongoing clinical challenge in targeting SI with currently available (online) interventions and emphasise the need for additional research examining efficacy of (novel) interventions targeting SI.

Our secondary analysis suggests that the effect of the GAF-ID intervention on SI at week 24 was mediated via depressive symptoms at week 10. This finding could suggest that an online BA intervention, such as GAF-ID, might be associated with alleviation of SI over time through targeting (other) depressive symptoms, which is in line with observations from clinical practice. However, this finding should be interpreted with caution since this was a secondary analysis. Further research is necessary to determine whether SI can be treated either directly or separately from depression, especially in light of the scarcity of effective interventions for SI in individuals with depression in LMICs.

Finally, we found differences in results between the PHQ-9 and IDS-SR in our primary analysis, before Bonferroni correction. The sensitivity analysis employing the IDS-SR to measure SI at week 24 showed initial support for mediation via BA at week 10, in favour of the GAF-ID group. This was in contrast to the mediation analysis employing the PHQ-9 to measure SI at week 24, which found no support for mediation via BA at week 10. The preliminary differences in results between the PHQ-9 and IDS-SR could possibly be explained by different psychometric properties, differences in item content, labelling or their construction. Although we found high concordance between the PHQ-9 and IDS-SR (online supplemental table 4), a concordance test may not be the best way to disentangle similarities or differences between these variables at such a fine-grained phenomenological level. Therefore, future research would need to assess SI using questionnaires primarily designed for this purpose,^31^ as well as applying more culturally sensitive methods. Currently, there is no specific information relating to the cultural sensitivity of the SI items of the PHQ-9 and IDS-SR.^14 17 19 32^ Our findings are too preliminary to draw conclusions relating to the cultural sensitivity of these items, especially since the different findings were no longer significant after Bonferroni correction.

### Strengths

The current study has a longitudinal study design with repeated measurements. Given the paucity of research on depression and SI in LMICs including Indonesia, this study extends current findings from HICs to Indonesia and could be of relevance to other countries in Southeast Asia. Moreover, the topic of the current study is imperative, since Indonesia has the highest number of unreported suicides in the world.^3 4^

### Limitations

Although this study extends previous research findings on the efficacy of an online BA intervention for SI, it is, however, not without limitations. First, the current study is a secondary analysis of an RCT and the analysis reported in this manuscript was not preregistered.^14^ Second, acute suicidality, defined as a suicide plan with preparatory behaviour was an exclusion criterium,^11^ which could have led to selection bias by not including the entire spectrum of suicidality, specifically the more severe end of the spectrum. Although this is an important limitation, only two participants were excluded due to acute suicidality, thereby limiting the risk of selection bias. Moreover, this approach is in line with the majority of research on suicidality, given the practical and ethical concerns of including patients with acute suicidality in RCTs.^33^ Third, we assessed SI using a single item from self-reported questionnaires. In addition, PHQ-9 item 9 and IDS-SR item 18 were recoded as a dichotomous variable due to lower numbers on higher scores of the items, which resulted in insufficient power to employ ordinal logistic regression models (online supplemental file, page 1). Previous literature has suggested that the use of a single item or a dimensional factor derived from a depression scale might be a valid approach to assess SI.^6 20 21 34–36^ However, given that SI exists on a continuum and is often characterised by a waxing and waning course, the approach of assessing SI in a dichotomised form has limitations. Nevertheless, the reporting of SI also needs to be seen in light of the cultural, religious and political context of Indonesia, where a religious taboo has long stigmatised SI and SB,^3^ which could have possibly led to lower scores on related questionnaire items. It would however be beneficial for future research to assess SI by using validated questionnaires primarily designed for this purpose.^31 37^ Fourth, there could have been parameter bias in the mediation analysis due to our relatively small sample size (n=313). Therefore, the proportion mediated was not calculated. Also, the PHQ-9 was measured biweekly (at baseline, weeks 2, 4, 6, 8 and 10), while the IDS-SR30 was only measured at baseline and week 10.^14^ Finally, since a between-group design does not control for variation among individual subjects, it would have been of interest to study whether within-person changes in BA or depressive symptoms mediated the intervention’s impact on SI. Therefore, we recommend to design future research that will allow a within-person analysis.

## Conclusions

The online GAF-ID intervention was not superior in reducing SI compared with online minimal PE in individuals with depression in Indonesia. In addition, contrary to our primary hypothesis, the association between treatment condition and SI at week 24 was not mediated via BA at week 10. This study contributes to the knowledge on online BA interventions for SI in the context of depression in Indonesia and supports the need for further research on the efficacy of psychological treatments for SI in the Southeast Asian context.

10.1136/bmjment-2023-300918.supp2 Supplementary data

## Data Availability

Data are available on reasonable request. The datasets presented in this article are not readily available because legal approval of the university is needed before raw data can be made available. Requests to access the datasets should be directed to c.b.heuschen@amsterdamumc.nl.
