# Supplementary material for: Internet-based behavioural activation therapy versus online psychoeducation for self-reported suicidal ideation in individuals with depression in Indonesia: a secondary analysis of an RCT
Source: BMJ Ment Health. 2024 Feb 20;27(1):e300918. doi: 10.1136/bmjment-2023-300918 (PMC10882357; doi:10.1136/bmjment-2023-300918)
Supplement: Supplementary data [file bmjment-2023-300918supp001.pdf]

Supplemental material

Operationalization of self-reported suicidal ideation as dichotomous variable

We conducted an additional ordinal logistic regression analysis, which showed that the expected log odds ratio decreased by 0·23 (95% CI (-0·81, 0·36)) with each category of the dependent variable (Y). This difference was not significant between treatment groups (p =0·45), and illustrates that the association between treatment allocation and suicidal ideation was not driven by the ordinal ranking of the SI item. However, these results should be interpreted with caution, given the small cell count in the more severe categories of PHQ-9 item 9. In line, the binary logistic regression analysis showed no significant differences between treatment groups (OR =0·84, 95% CI (0·47, 1·52), p =0·56).

Supplemental table 1. Suicidal ideation

IDS-SR item 18	Treatment group (GAF-ID)	Control group (PE)	OR	95% CI	p
At baseline	114 (72%)	105 (68%)	1·18	0·73, 1·92	0·50
At post intervention	53 (44%)	76 (52%)	0·72	0·44, 1·17	0·18
At 6 months follow up	28 (25%)	58 (40%)	0·49	0·28, 0·84	0·01*

IDS-SR item 18: thoughts of my own death or suicide (0/1/2/3) recoded into two categories: no suicidal ideation (score of 0) versus the presence of suicidal ideation (scores ≥1).

Supplemental table 2. Overview of temporally ordered mediation models (n = 313)

Mediator week 10	Outcome week 24	Direct effect				Indirect effect			
		Estimate	SE	95% CI LL - UL	P	Estimate	SE	95% CI LL - UL	p
BADS-SF	PHQ-9 item 9	-0·00	0·05	-0·10, 0·10	0·96	-0·03	0·01	-0·05, 0·00	0·07
SUM PHQ-9	PHQ-9 item 9	0·01	0·05	-0·08, 0·11	0·82	-0·04	0·02	-0·07, -0·01	0·01*
BADS-SF	IDS-SR item 18	-0·11	0·06	-0·23, 0·09	0·06	-0·03	0·02	-0·06, -0·00	0·03
SUM IDS	IDS-SR item 18	-0·11	0·06	-0·23, 0·00	0·05*	-0·03	0·01	-0·06, -0·00	0·02

Direct effect: path c'; indirect effect: shown as indirect effect coefficient (function of the compound pathway *ab*).

SUM PHQ-9 and SUM IDS; sum scores of PHQ-9 and the Indonesian IDS-SR were created while excluding the item related to suicidal ideation (PHQ-9 item 9, IDS-SR item 18 respectively) from the total score in order to test suicidal symptom scores independently from total scores of depressive symptomatology.

Supplemental table 3. Post-hoc analyses (n = 313)

**Mediator (M)** **BADS-SF**	**Outcome (Y)** **PHQ-9 item 9**	**Direct effect**				**Indirect effect**			
		Estimate	SE	95% CI LL - UL	P	Estimate	SE	95% CI LL - UL	p
week 2	Week 10	-0·09	0·06	-0·20, 0·03	0·14	-0·01	0·01	-0·03, 0·01	0·33
Week 4	Week 10	-0·07	0·06	-0·19, 0·05	0·24	-0·03	0·01	-0·05, 0·00	0·08
Week 6	Week 10	-0·05	0·06	-0·18, 0·06	0·32	-0·04	0·02	-0·07, -0·00	0·03
Week 8	Week 10	-0·06	0·06	-0·17, 0·05	0·31	-0·04	0·02	-0·07, -0·00	0·03

Direct effect: path c'; indirect effect: shown as indirect effect coefficient (function of the compound pathway *ab*).

Supplemental table 4. Concordance of PHQ-9 item 9 and IDS-SR item 18

	IDS-SR item 18	χ2	df	*p*	Symmetry *p*
**PHQ-9 item 9**					
Baseline	94%	139·60	1	0·00	0·00
Posttest	86%	96·00	1	0·00	0·00
Six months follow up	88%	102·81	1	0·00	0·00

PHQ-9 item 9: thoughts that you would be better off dead: 0/1/2/3
IDS-SR item 18: thoughts of my own death or suicide: 0/1/2/3

Supplemental figure 1. Behavioural activation

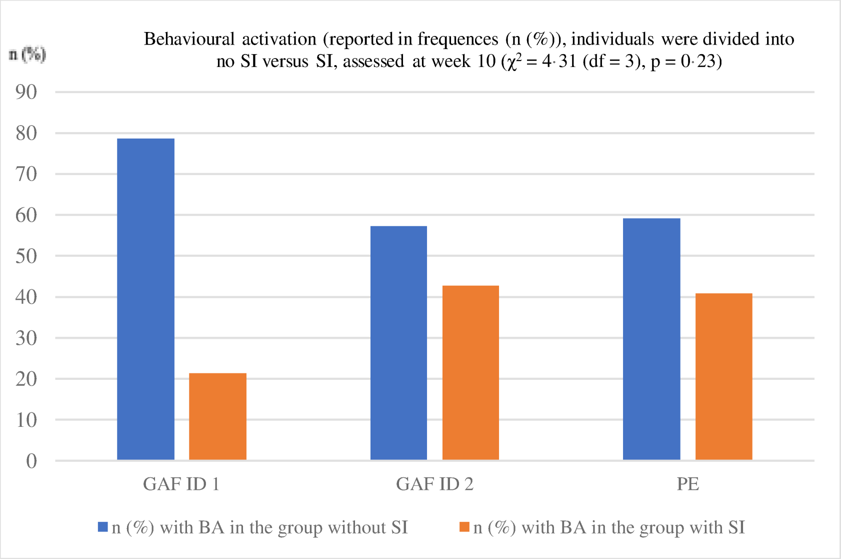

SI: suicidal ideation; GAF ID 1: BADS-SF score ≥22; GAF ID 2: BADS-SF score <22.

* The GAF-ID group was divided into two groups, based on the median BADS-SF score in the total group (<22 or 22 and above).
