# Supplementary material for: Internet-based behavioural activation therapy versus online psychoeducation for self-reported suicidal ideation in individuals with depression in Indonesia: a secondary analysis of an RCT
Source: BMJ Ment Health. 2024 Feb 20;27(1):e300918. doi: 10.1136/bmjment-2023-300918 (PMC10882357; doi:10.1136/bmjment-2023-300918)
Supplement: Supplementary data [file bmjment-2023-300918supp002.pdf]

Arjadi et al. Trials (2016) 17:455
DOI 10.1186/s13063-016-1577-9

Trials

STUDY PROTOCOL Open Access

Guided Act and Feel Indonesia (GAF-ID) – Internet-based behavioral activation intervention for depression in Indonesia: study protocol for a randomized controlled trial

Retha Arjadi[1,2], Maaike H. Nauta[1], Willem F. Scholte[3,4], Steven D. Hollon[5], Neerja Chowdhary[6], Angela O. Suryani[2] and Claudi L. H. Bockting[1,7*]

Abstract

Background: Depression is a leading cause of disease burden across the world. However, in low-middle income countries (LMICs), access to mental health services is severely limited because of the insufficient number of mental health professionals available. The WHO initiated the Mental Health Gap Action Program (mhGAP) aiming to provide a coherent strategy for closing the gap between what is urgently needed and what is available in LMICs. Internet-based treatment is a promising strategy that can be made available to a large number of people now that Internet access is increasing rapidly throughout the world. The present study will investigate whether such an Internet-based treatment for depression is effective in Indonesia.

Methods: An Internet-based behavioral activation treatment, with support by lay counselors who will provide online feedback on the assignments and supportive phone contact to encourage participants to work in the program (Guided Act and Feel Indonesia/GAF-ID), is compared to an online-delivered minimal psychoeducation without any support (psychoeducation/PE). Initial assessment for inclusion is based on a Patient Health Questionnaire-9 (PHQ-9) score of at least 10 and meeting criteria for major depressive disorder or persistent depressive disorder as assessed using the Structured Clinical Interview for DSM-5 (SCID-5). Participants with depression ($N = 312$) will be recruited and randomly assigned to GAF-ID or PE. Overall assessments will be done at baseline, post intervention (10 weeks from baseline) and follow-ups (3 months and 6 months from baseline). The primary outcome is the reduction of depression symptoms as measured by the PHQ-9 after 10 weeks from baseline.

Discussion: To our knowledge, this is the first study in Indonesia that examines the effectiveness of an Internet-based intervention for depression in a randomized controlled trial. The hope is that it can serve as a starting point for bridging the mental health gap in Indonesia and other LMICs.

Trial registration: Nederlands Trial Register (www.trialregister.nl): NTR5920, registered on 1 July 2016.

Keywords: Internet-based intervention, Online therapy, Behavioral activation, Depression, Lay counseling, Psychological interventions, Indonesia, Developing country, Low and middle income country, LMIC

* Correspondence: c.l.h.bockting@uu.nl
[1]Department of Clinical Psychology and Experimental Psychopathology, University of Groningen, Groningen, The Netherlands
[7]Department of Clinical Psychology, Utrecht University, Heidelberglaan 1, 3584 CS Utrecht, The Netherlands
Full list of author information is available at the end of the article

© 2016 The Author(s). **Open Access** This article is distributed under the terms of the Creative Commons Attribution 4.0 International License (http://creativecommons.org/licenses/by/4.0/), which permits unrestricted use, distribution, and reproduction in any medium, provided you give appropriate credit to the original author(s) and the source, provide a link to the Creative Commons license, and indicate if changes were made. The Creative Commons Public Domain Dedication waiver (http://creativecommons.org/publicdomain/zero/1.0/) applies to the data made available in this article, unless otherwise stated.

Background

Depression is a leading cause of disease burden and health care costs across the world, with approximately 350 million people affected, equivalent to 5 % of the world population [1]. In 2006, depression was predicted to be the second largest cause of disease-related disability by 2030 [2]. There are many negative consequences of depression, such as impairment of an individual's home and work functioning, an increase of comorbidity from other health problems, and an increase in mortality, partly due to suicide [3, 4]. Depression undermines the quality of life of those afflicted, has an emotional and financial impact on their families, and has an economic impact on society at large [4].

In almost all countries, the prevalence of depression is associated with the availability of treatment. However, the ability of low-middle income countries (LMICs) to provide access to mental health care for depression is severely limited. A large epidemiological study found that 35.5–50.3 % of the people with severe mental disorders in high-income countries (HICs) received no treatment at all in the previous year; meanwhile, the number reached 76.3–85.4 % in LMICs [5].

The World Health Organization (WHO) initiated the mental health Gap Action Program (mhGAP) aiming to provide a coherent strategy for closing the gap between what is urgently needed and what is available, with a final goal of reducing the burden of mental disorders worldwide, especially in LMICs like Indonesia, one of the countries identified for intensified support on the WHO mhGAP country list [1]. A recent study on basic health indicators in Indonesia found that the prevalence of depression and anxiety based on self-report is estimated at 6 % in adolescents and adults (aged 14 years or older), lower than in most other countries, but still equivalent to approximately 14 million people [6].

Depression can be treated using a Behavioral Activation (BA) intervention which is known to be a simple yet effective treatment for acute depression [7]. It uses behavioral strategies such as activity scheduling and re-engagement in pleasurable and rewarding activities to restore positive mood [8]. BA has been widely investigated and is applicable to a broad range of populations, including adolescents [9], normal-aged adults [10], and older adults [11]. It also has been found to be effective for the Latino population in the United States [10] and in one LMIC, India [12].

Despite the evidence, delivering BA in a LMIC like Indonesia is rather challenging. The main challenges faced by LMICs in general are low budgets for mental health [13] and the lack of trained professionals outside of urban areas [1, 14–16]. As in many other LMICs, the availability of psychological interventions in Indonesia is very limited and largely disproportionate between mental health professionals and inhabitants. The proportion of 2.91 mental health professionals for 100,000 inhabitants in Indonesia is even lower than the minimum proportion of 3.33 per 100,000 [1, 13]. Moreover, in Indonesia, apart from medication, mental health care is usually not covered by medical insurance and is expensive for most people. To counter this, one strategy could be to provide an Internet-based intervention. This has been shown to be effective for depression from studies conducted in HICs [17–19] as a relatively low-cost mental health intervention that can be widely distributed, but has been rarely studied in LMICs [20].

A report from 2014 revealed that 88 million people in Indonesia (34.9 % of the population) used the Internet [21] and that number is expected to increase to 120 million people (50 % of the population) by 2018 [22]. The Internet is also already used for online support groups of people with mental health problems in Indonesia, such as for mood disorders and schizophrenia. Therefore, based on the evidence of its effectiveness in HICs and given the limited access to mental health professionals along with the increase of Internet usage in Indonesia, Internet-based intervention might be a promising treatment for depression in Indonesia.

Internet-based interventions have been found to be particularly effective if therapist support is provided [19]. However, it is yet unclear whether highly qualified therapists are necessary to provide this support. In fact, there are good examples that face-to-face BA can be delivered by trained nonprofessionals or lay counselors in both a HIC [23] and a LMIC [12]. Moreover, other than BA, previous studies in other LMICs, Uganda, India, and Pakistan have indicated that lay counselors could successfully deliver effective face-to-face treatments for depression [24–27].

For the current study, we adapted a Dutch Internet-based BA intervention program for use in Indonesia [28]. The participants in this study will be supported by lay counselors under the supervision of a small number of licensed clinical psychologists. We will include lay counselors who do not have any professional clinical experiences and we will train them for the purpose of this particular research. We plan to study the effectiveness of this online Internet-based BA called "Guided Act and Feel Indonesia" (GAF-ID) as guided by lay counselors. GAF-ID is delivered using a website where the participants are able to access the intervention content. Like any other Internet-based psychological interventions, GAF-ID consists of information presented in an attractive visual format, step-by-step instructions, and a structured format of weekly assignments.

This study will compare the effectiveness of GAF-ID, an Internet-based BA with support by lay counselors, to a control group providing online-delivered minimal

Supplemental material BMJ Publishing Group Limited (BMJ) disclaims all liability and responsibility arising from any reliance placed on this supplemental material which has been supplied by the author(s) *BMJ Ment Health*

psychoeducation (PE) without support in the treatment of individuals with a depressive disorder. Our hypothesis is that the GAF-ID will be superior to PE in reducing depressive symptoms in participants with a depressive disorder.

Methods

This study has been designed according to the Standard Protocol Items: Recommendations for Interventional Trials (SPIRIT) statement [29]. Figure 1 presents a flow diagram of the study design, and the SPIRIT checklist is provided as Additional file 1.

Participants

Recruitment

We aim to recruit 312 participants for this study from the community through advertisements, via the mass media (banners placed in various related websites and places, newspapers and magazines), social media (online groups, pages, and forums related to mental health issues), and referral from mental health institutions or mental health professionals. The potential participants will be able to access information regarding the trial on our study website: www.actandfeel.com and they can fill in the Patient Health Questionnaire-9 (PHQ-9) [30] for screening purpose on a linked online survey system, the Qualtrics website. If they have scores of at least 10, we will make sure that they understand all aspects of the trial and invite them or their legal guardian to fill in a written informed consent to provide their sociodemographic information and join a clinical interview using the Structured Clinical Interview for the *Diagnostic and Statistical Manual of Mental Disorders*, *fifth edition* (DSM-5) (SCID-5) [31] through a phone call. If they meet all inclusion and none of the exclusion criteria for the study, they are eligible to join the study. The participants will also be informed that each of them who is participating until the last follow-up assessment (6 months from baseline) will receive a monetary incentive, irrelevant of the group to which they belong.

Inclusion criteria

Participants will be included when they meet these following criteria: (1) meet the cutoff score of at least 10 on the PHQ-9 [30], (2) meet the criteria for a diagnosis of major depressive disorder or persistent depressive disorder on the SCID-5 [31], (3) are aged 16 years or older, (4) are proficient in the Indonesian language, and (5) have fluency to use the Internet. There is no restriction to join the study for participants who have the comorbidity of anxiety as measured using the Fear Questionnaire [32] or trauma- and stressor-related disorders as reported on the SCID-5 interview [31].

Exclusion criteria

A potential participant who meets any of the following criteria (as indicated by the SCID-5 [31] will be excluded from participation in this study: (1) current or previous manic or hypomanic episode, (2) current or previous psychotic disorder, (3) current substance use disorder, and (4) acute suicidality. They will then be contacted by a clinical psychologist and provided with appropriate referral information for further treatment. We will also exclude those who (5) currently follow a weekly or more

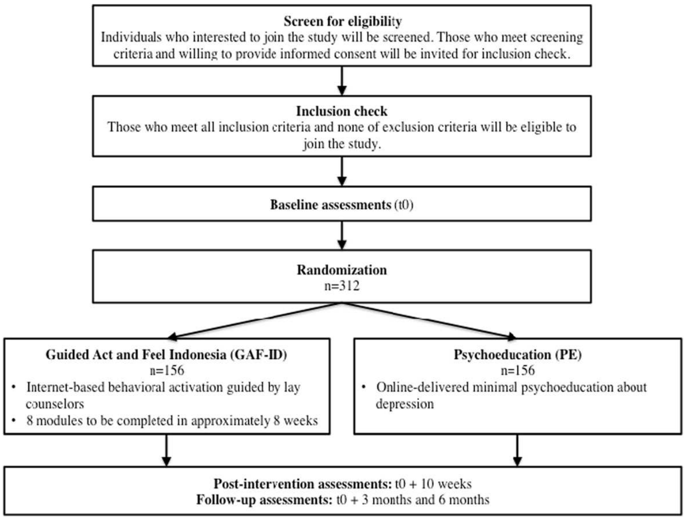

Fig. 1 Flow diagram

intensive psychological intervention (non-medication) for mental health complaints.

Study design

We will conduct a randomized controlled trial (RCT) in Indonesia with participants being assigned to either an intervention group or a control group. The intervention group will be given the Internet-based BA intervention, supported by lay counselors, called "Guided Act and Feel Indonesia" (GAF-ID). Those in the control group will receive online-delivered minimal psychoeducation (PE) without support. The GAF-ID will be delivered via one secure online platform, at the following address: www.actandfeelindonesia.com. This platform is built by an independent professional intervention website developer in The Netherlands. Meanwhile, the PE will be delivered via a different website: www.actandfeel.com. Each participant will be provided with a personal username and password to either GAF-ID or PE, based on the group to which they are randomized.

Intervention: Guided Act and Feel Indonesia (GAF-ID)

The content of the GAF-ID program is based on the face-to-face BA intervention [7] and on the Dutch online BA intervention, called "Act and Feel" [28]. The GAF-ID is offered in a secure online environment and consists of a series of eight structured modules that can be completed over 8 weeks.

BA is based on the behavioral theory stating that depression is a consequence of low rates of response-contingent positive reinforcement. Therefore, BA concentrates on activating individuals to increase contact with potential reinforcers [7]. The main focus of BA is to increase potential pleasurable activities that are preplanned and mood-independent in order to enhance mood and thereby overcome depression in the long term. In BA, the participants are first taught to observe their daily mood and behaviors/activities using daily activity monitoring. They are then asked to schedule activities and increase their daily pleasurable activities. Moreover, specific attention will be given to the role of avoidance in depression and how to break the pattern from predominantly reactive behavior towards proactive behavior [7, 8, 33].

There are some adjustments made from the original "Act and Feel" to make the content of the GAF-ID more accessible for the Indonesian population who may not have very good Internet connections: videos of therapists and case examples were replaced by a series of illustrative pictures and cartoons in an effort to compensate for the low-speed Internet connections in Indonesia. We also adjusted the examples of the assignments and the stories of previous participants' experiences to make them more relevant to Indonesian culture. All adjustments were made based on discussion within the research team, on consultation with clinical psychologists in Indonesia, and on suggestions from lay counselors who support this project. The adjusted version of the program was tested several times in a pilot testing including persons with different characteristics (age, sex, education, occupation, and place of abode) to check the usability, readability and acceptability of the content. More adjustments were made based on the pilot testing results, such as shortened and clarified texts, jargon replaced with easier terms, and the addition of some illustrations to make the program more attractive. However, the sequence and basic content of the eight modules used in the GAF-ID is the same as in the original Dutch version.

Over the eight modules, the main elements are as follows: understanding the basic background of BA and psychoeducation about depression, monitoring mood and behavioral activities, expanding potential mood-independent pleasurable activities, recognizing and overcoming difficulties with expanding activities, realizing the impact of avoidance behaviors, and building a prevention of relapse strategy. The GAF-ID also provides automatized feedback and automatically graphs mood self-ratings to monitor progress for each participant. Like the original face-to-face BA, the GAF-ID modules follow a fixed structure. Each module starts with a rationale for that module and is followed by specific assignments. Each module can be completed in approximately 30–45 min excluding time to complete assignments. However, there is no time restriction.

Each participant is assigned to a personal lay counselor (supervised by a clinical psychologist) to support the participant in following the online program. Over the course of the program, the lay counselors provide brief feedback online regarding the assignments for each of the eight modules via the online program. In addition, the lay counselors will make brief contact via phone calls to reinforce and encourage participants to work in the online program (weekly during the first 4 weeks and at weeks 6 and 8) in order to enhance adherence to the program and to prevent attrition. The calls do not take more than 20 min each and there is no option for a face-to-face contact.

The GAF-ID program is equipped with a messaging facility that will be used as the main medium of communication between the participants and their lay counselors. The lay counselors log into the GAF-ID program regularly to see the assignments that have been undertaken by the participants and to provide feedback consistent with the BA rationale. If a module has been completed satisfactorily, the lay counselor sends a short message through the GAF-ID program with feedback on the previous module and encourages the participants to engage in the next module. If the participants cannot

finish the assignments or seem to not understand the instructions, they can send a message to their lay counselors, and the lay counselors will provide assistance. Further, the lay counselors can see the log-in history of the participants they have assisted, and can send reminders via email and text message to those participants who do not log in to the program each week. Phone calls will follow if the participant fails to reinitiate the intervention after one more week.

All lay counselors will work under the supervision of licensed clinical psychologists and regularly report on the participant's progress to them as their supervisors. The lay counselors present their cases to their supervisors and address concerns in regular weekly supervision.

Control condition: online-delivered minimal psychoeducation (PE)

In the PE group, as an active comparison condition, the minimal PE is presented as a short, online leaflet consisting of basic information about depression and basic tips on how it can be addressed, representing information that can be easily and freely accessed online outside of this program. Participants in the PE group will receive neither assistance from lay counselors nor from clinical psychologists, but provided with the same assessments as the intervention group.

Support

Lay counselors

We plan to recruit 20–30 persons to serve as lay counselors for this study. Our lay counselors meet the following criteria: (1) age between 20 and 40 years with no restriction on gender, (2) minimum senior high school education, (3) willing to participate fully during the trial process, (4) no professional background as a mental health specialist, and (5) willing to participate in the training for lay counselor in this study.

The focus of the lay counselors' support is to help each participant follow the GAF-ID program by (1) explaining to the participants how GAF-ID works, (2) providing technical assistance for participants, (3) providing short feedback to each of the completed modules, and (4) reminding participants to complete each online module. The lay counselors will not provide any additional counseling.

All lay counselors receive 2 days of intensive training during which all features of the Internet-based BA are discussed and role-plays are conducted. Other technical issues addressed during the training include how to handle technical problems that may arise, how to handle participants with low motivation, and how to monitor suicidality or other serious deteriorations during the intervention. They will receive printed training modules to help them do their tasks during the trial.

To promote treatment integrity and consistency, we provided, apart from the 2 days of training, a treatment guidance protocol, a weekly structured support checklist, a participant's short progress report template, and regular supervision by a clinical psychologist. In order to elicit and to monitor adherence, the lay counselors will fill out a weekly structured checklist on the support that they have given to each participant. To assure integrity and consistency further, the lay counselors will also be asked to write a regular short progress report on each participant. They will give both the checklist and the report to the clinical psychologists who supervised them. The clinical psychologists will then provide necessary feedback and consultations.

Clinical psychologists

We plan to recruit 10–15 clinical psychologists to provide clinical supervision to the lay counselors in this trial. Each clinical psychologist will supervise three to five lay counselors. All clinical psychologists will be graduates from a formal clinical psychology program and need to be licensed as clinical psychologists in Indonesia. The clinical psychologists will receive a weekly structured support checklist and a short report of participants' progress from the lay counselors under their supervision that they can discuss together in the regular supervision meetings.

Similar to what is provided for the lay counselors, the clinical psychologists will be provided with 2 days of intensive training on the GAF-ID program and a printed version of the training module. The training is given to make sure that the clinical psychologists are familiar with the program and can provide necessary supervision to the lay counselors.

The clinical psychologists will only have contact with participants in case of a suicidal crisis and other serious deteriorations, through phone calls. They will be equipped with the standardized procedure on when and how they should do suicide risk assessment and handle serious participant deteriorations within this trial.

Outcome assessments

There will be several points of assessment conducted during this study: baseline, post intervention (10 weeks from baseline), and follow-ups (3 months and 6 months from baseline). There will also be a biweekly (once every 2 weeks) assessment in between. All self-report assessments will be conducted through the Qualtrics website, with a private link delivered to the participant's personal email at each time point. See Table 1 for the details.

Primary outcome

The primary outcome is self-reported depressive symptoms based on the Patient Health Questionnaire-9

Table 1 Assessments

Measures	Description	Baseline (t0)	2 weeks	4 weeks	6 weeks	8 weeks	10 weeks (post intervention)	3 months (follow-up)	6 months (follow-up)
Primary measure									
PHQ-9	Depression symptoms level	+	+	+	+	+	+	+	+
Secondary measures									
SCID-5 (interview)	Current depressive disorder (major depressive disorder and persistent depressive disorder)	+					+		
IDS-SR	Depression symptoms	+					+	+	+
FQ	Fear and avoidance	+					+	+	+
MSPSS	Perceived social support	+					+	+	+
WHOQOL-BREF	Quality of life	+					+	+	+
Potential mediators and moderators									
VAS	General mood condition	+	+	+	+	+	+	+	+
PANAS	Positive and negative affects	+	+	+	+	+	+	+	+
BADS-SF	Behavioral activation	+	+	+	+	+	+	+	+
Life-events	Life events	+							
SCID-5 (interview)	• History of depressive disorders (major depressive disorder or persistent depressive disorder)<br>• Trauma- and stressor-related disorders	+							
Childhood trauma (interview)	History of childhood trauma	+							
Additional measures									
MEIM	Multigroup ethnic identity measure	+							
Demographics	Sociodemographic characteristics	+							
Clinical information	Information related to clinical conditions	+							

BADS-SF Behavioral Activation for Depression Scale Short Form, *FQ* Fear Questionnaire, *IDS-SR* Inventory of Depressive Symptomatology Self-Report, *MEIM* Multigroup Ethnic Identity Measure, *MSPSS* Multidimensional Scale of Perceived Social Support, *PANAS* Positive and Negative Affect Scale, *PHQ-9* Patient Health Questionnaire-9, *SCID-5* Structured Clinical Interview for DSM-5, *VAS* Visual Analogue Scale, *WHOQOL-BREF* The brief version of the WHO Quality Of Life

The time frames of 2 weeks, 4 weeks, 6 weeks, 8 weeks, 10 weeks, 3 months, and 6 months are counted from baseline (applied in both groups)

(PHQ-9) [30] at post intervention (10 weeks from baseline). However, overall during the study, it will be administered at baseline, post intervention (10 weeks from baseline), follow-ups (3 months and 6 months from baseline), as well as biweekly during the 8-week intervention period.

Secondary outcomes

Secondary outcomes will include: (1) rate of remission/ recovery of depression (major depressive disorder or persistent depressive disorder) using the Structured Clinical Interview for DSM-5 (SCID-5) [31], (2) the Inventory of Depressive Symptomatology Self-Report (IDS-SR) [34, 35], (3) the Fear Questionnaire (FQ) [32], (4) the Multidimensional Scale of Perceived Social Support (MSPSS) [36], and (5) The brief version of the WHO Quality of Life (WHOQOL-BREF) [37].

Potential mediators and moderators

For potential moderators and mediators of the outcome, we will assess: (1) a Visual Analogue Scale (VAS) of mood (one-item mood scale) [38], (2) the Positive and Negative Affect Scale (PANAS) [39], (3) the Behavioral Activation for Depression Scale Short Form (BADS-SF) [40], (4) the Life-events scale [41], (5) history of depressive disorders (major depressive disorder and persistent depressive disorder) and trauma- and stressor-related disorders as assessed using the SCID-5 interview [31], and (6) our self-developed Childhood trauma.

Additional measures

For additional measures, since Indonesia is rich in cultural diversity, we will collect data on ethnic identity using the Multigroup Ethnic Identity Measure (MEIM) [42]. We will also examine sociodemographic characteristics (i.e. age, gender) and clinical information (i.e. depression attribution, psychiatric-related health care consumption) in each participant.

Main study hypotheses

That participants receiving the GAF-ID will have a lower level of depression symptoms on the PHQ-9 [30] at the main time point of measuring effectiveness after 10 weeks from baseline (post intervention) compared to participants in the PE group. The lower level of depression symptoms measured using the same tool is also expected to present in the GAF-ID arm relative to the PE arm at the 3-month and 6-month follow-ups.

Furthermore, the incidence of major depressive disorder and persistent depressive disorder measured using the SCID-5 [31] is expected to be lower in the GAF-ID group compared to the PE group at post intervention and follow-ups, along with lower depressive symptoms measured using the IDS-SR [34, 35], lower anxiety levels measured using the FQ [32], higher perceived social support as measured by the MSPSS [36], and higher quality of life as measured by WHOQOL-BREF [37].

All potential moderators are hypothesized to predict outcome measures, whereas potential mediators are supposed to underlie the reduction in depression symptoms. Moreover, ethnic identity, sociodemographic characteristics and clinical information will be examined as additional data.

Power calculation

Psychoeducation has been used as the comparator in some online and non-online depression treatment trials with effect sizes ranging from 0.03 to 0.85 [43–45]. Given the fact that we plan to perform this study in a unique target population (a LMIC), the statistical power was calculated to estimate a rather conservative small to medium effect size. The power is calculated to detect differences between two independent groups, in a two-sided test at alpha = 0.05 and a power of (1 – beta) = 0.80, for an effect size of 0.35 (small to medium effect size). Based on these parameters, we will need a sample of 260 participants. To compensate for the expected 20 % attrition we will need to include and randomize 312 participants at baseline.

Randomization and blinding

As participants are screened into the study, they will be randomized using a web-based program that was built for this trial. Randomization will be performed within in a permuted block design. The size of the blocks and the exact strata are not revealed in this design paper, so that the underlying algorithm remains unpredictable for the research assistants, but it is stated on the trial registration. This study is single-blind: the research assistants, who will be involved in conducting the clinical interviews after randomization, will be blind to the treatment condition and the participants will be asked not to reveal their treatment condition during the interview. Research assistants who perform the assessments are not involved in the intervention process and they will be asked to guess the treatment allocation per participant.

Analysis

Primary analysis

The primary analysis will be conducted on an intent-to-treat basis, including all participants randomized to the study regardless of treatment adherence or attrition or the completion of outcome assessments. The PHQ-9 [30] will serve as the primary outcome measure with the main time point of effectiveness at post intervention (10 weeks from baseline). Prior to the effect analyses the baseline comparability of the two groups in terms of prognostic variable distributions will be checked. If,

despite randomization important differences exist, these variables will be adjusted for in the analyses by their inclusion as covariates.

Each repeated outcome measure will be analyzed as a dependent variable using linear mixed models for fixed (treatment) and random effects models (participant) for independent variables. These models are superior for the analysis of longitudinally correlated data (within subjects) and can optimally deal with missing values, i.e. they prevent complete-case bias by incorporating all available data. In these analyses, a treatment × time interaction term will represent the effect of the intervention on the change of the outcome variable over time. Effect modification of treatment by potential moderators will be studied by including interaction terms of treatment times the moderator variable and testing their statistical significance. Potential mediation of the effect of treatment will be studied using the mediation model proposed by Preacher and Hayes [46]. All effects parameters will be supplied with a 95 % confidence interval. The two-sided significance level will be set at $p = 0.05$.

Other analyses

All other measures will be analyzed as other analyses. The analyses will also be conducted on the participants who complete treatment, with completion being defined as finishing at least five of the eight modules of the GAF-ID. Potential selection bias associated with completion will be corrected by including unequally distributed baseline prognostic variables as independent variables in the models. We will also perform responder analysis to investigate the number of patients who recover from depressive disorder in the intervention group and the control group based on the SCID-5 interview [31].

Additionally, we also are interested in further analysing ethnic identity that might have specific impact on depression among Indonesians. Culture is one of the factors that has been reported to contribute to the experience and symptoms of depression [47]. For this purpose, the ethnic background from each participant will be reported, and particular participants with a strong ethnic identity shown by a high score on the MEIM [42] will further be profiled on their depression symptoms.

Suicidality and serious deterioration

Suicidal thoughts and tendencies will be assessed biweekly in both conditions using the PHQ-9 [30] during the study. Participants scoring at least 2 on the suicide item "Thoughts that you would be better off dead or of hurting yourself in some way" twice in a row will be contacted by a clinical psychologist via a phone call for suicide risk assessment. Moreover, serious deterioration will be detected via the biweekly measures: in case an increase in depression level measured by the PHQ-9 [30] occurs (score increase by at least 5 points with a total score of at least 15 points) and this increase is still present or even more pronounced 2 weeks later, participants will be contacted by a trained research assistant for an IDS-C interview [34] via a phone call. If the score is at least 24, then a SCID-5 interview [31] on depression and screening for other potential deteriorations will follow. A full SCID-5 interview [31] for specific section will be followed if the participants answer "yes" to either of the screening questions.

For both treatment conditions, in case of severe suicidality and serious deterioration, more intensive treatment may be required and the participant may need to be referred to a mental health professional. They will be provided with appropriate referral information when necessary. However, all participants will be asked to continue with the intervention and the assessments.

At post intervention (10 weeks from baseline), as an outcome assessment, we will conduct the SCID-5 interview [31] on depression. At this point, we will also use the SCID-5 [31] to screen for other deteriorations in all participants, followed by the full section interview if the participants answer "yes" to either of the screening questions. The same referral procedure will be applied if any serious deterioration is found.

Discussion

To our knowledge, this will be the first study in Indonesia that investigates the effectiveness of an Internet-based psychological intervention for depression in a RCT. This trial offers new avenues for mental health treatments in LMICs, using Internet and additional support via an online voice call or phone call by lay counselors. If this intervention is effective, this treatment strategy can widen access for mental health services to many people from different locations in Indonesia.

Furthermore, it has been reported that the effect of an Internet-based cognitive-behavioral intervention for depression is still effective until 12 months after the intervention termination [45]. In this study, with assessments at post intervention (10 weeks from baseline) and follow-ups (3 months and 6 months from baseline), we will be able to assess the impact of this Internet-based behavioral activation intervention on depression in the longer term, including the impact on secondary outcomes. This study might be a starting point for showing the potential of using Internet-based intervention, and Internet-based BA in particular, to reduce the gap in mental health services availability in LMICs.

We also will try to identify factors that might contribute to depression and its recovery by collecting data regarding common comorbidity and other factors related to depression. It will provide information about the

factors that require more attention in tailoring the Internet-based therapy program and implementing such an indicated intervention for depression in Indonesia.

Trial status

Recruitment of the participants starts in September 2016. All participants are expected to finish their 6-month follow-up assessment by the end of 2017.

Additional file

Additional file 1: The SPIRIT checklist. (DOC 122 kb)

Abbreviations

BA: Behavioral activation; BADS-SF: Behavioral activation for depression scale short form; FQ: Fear questionnaire; GAF-ID: Guided Act and Feel Indonesia; HIC: High-income country; IDS-SR: Inventory of depressive symptomatology Self-Report; LMIC: Low-middle income country; MEIM: Multigroup ethnic identity Measure; MSPSS: Multidimensional Scale of Perceived Social Support; PANAS: Positive and Negative Affect Scale; PE: Psychoeducation; PHQ-9: Patient Health Questionnaire-9; RCT: Randomized controlled trial; SCID-5: Structured Clinical Interview for DSM-5; VAS: Visual analogue scale; WHOQOL-BREF: The brief version of the WHO Quality of Life

Acknowledgements

We would like to thank Prof. Vikram Patel for giving us valuable guidance on conducting clinical trials in a LIMC, Dr. Huibert Burger for his helpful advice on the statistical analysis, and Dr. Jap Tji Beng together with Dr. Sri Tiatri for assisting us in the ethical review application. We also would like to express our gratitude to the Indonesia Endowment Fund for Education (Lembaga Pengelola Dana Pendidikan), Ministry of Finance, Republic of Indonesia for making this study possible by providing a PhD scholarship along with research funding for the first author. We would also like to acknowledge the University of Groningen for its general funding support.

Funding

This study is funded by the Indonesia Endowment Fund for Education (Lembaga Pengelola Dana Pendidikan), Ministry of Finance, Republic of Indonesia which provided a PhD scholarship and research funding for the first author. In addition, the University of Groningen also provided general funding support.

Availability of data and materials

Additional file 1: The SPIRIT checklist.

Authors' contributions

RA, MN, SH, and CB worked on the original idea of this study and developed the design. SH gave advice on the details of each part of the study. WS provided assistance on the implementation of the study in a LIMC. NC and WS gave input on the details of engaging, recruiting and training lay counselors in the study. AS gave suggestions related to ethnicity measures in the study and provided recommendations on how to run the study in Indonesia. All authors contributed to this trial protocol paper and approved of the final version of the manuscript.

Competing interests

CB is the creator of the Internet-based BA used in this study. The authors declare that they have no competing interests.

Consent for publication

Consent for publication forms a specific part of the consent for participation. It has been obtained from all participants or their legal guardians.

Ethics approval and consent to participate

The Tarumanagara University Human Research Ethics Committee (TUHREC), Jakarta, Indonesia approved the trial protocol (project number: PPZ20152002). The TUHREC will ensure that the study is conducted within appropriate human research ethical guidelines and has a proper data management system. A written report will be provided by the researchers for the Ethical Committee whenever necessary. Consent for participation has been obtained from all participants or their legal guardians.

Author details

[1]Department of Clinical Psychology and Experimental Psychopathology, University of Groningen, Groningen, The Netherlands. [2]Faculty of Psychology, Atma Jaya Catholic University of Indonesia, Jakarta, Indonesia. [3]Academic Medical Center, Department of Psychiatry, University of Amsterdam, Amsterdam, The Netherlands. [4]Equator Foundation, Diemen, The Netherlands. [5]Department of Psychology, Vanderbilt University, Nashville, TN, USA. [6]International Medical Corps, Washington DC, USA. [7]Department of Clinical Psychology, Utrecht University, Heidelberglaan 1, 3584 CS Utrecht, The Netherlands.

Received: 24 July 2016 Accepted: 27 August 2016
Published online: 17 September 2016

References

1. World Health Organization. mhGAP: mental health Gap Action Program: scaling up care for mental, neurological, and substance use disorders. Geneva: WHO Press; 2008.
2. Mathers CD, Loncar D. Projections of global mortality and burden of disease from 2002 to 2030. PLoS Med. 2006;3:e442.
3. Herring MP, Puetz TW, O'Connor PJ, Dishman RK. Effect of exercise training on depressive symptoms among patients with a chronic illness: a systematic review and meta-analysis of randomized controlled trials. Arch Intern Med. 2012;172:101–11.
4. Lepiné JP, Briley M. The increasing burden of depression. Neuropsychiatr Dis Treat. 2011;7:3–7.
5. Demyttenaere K, Bruffaerts R, Posada-Villa J, Gasquet I, Kovess V, Lepine JP, et al. Prevalence, severity, and unmet need for treatment of mental disorders in the world health organization world mental health surveys. JAMA. 2004;291:2581–90.
6. Badan Penelitian dan Pengembangan Kesehatan Departemen Kesehatan Republik Indonesia. Riset kesehatan dasar 2013 (Basic Health Research 2013). Jakarta: Badan Penelitian dan Pengembangan Kesehatan Departemen Kesehatan Republik Indonesia; 2013.
7. Lewinsohn PM. A behavioral approach to depression. In: Coyne JC, editor. Essential papers on depression. New York: New York University Press; 1985. p. 150–80.
8. Martell CR, Dimidjian S, Herman-Dunn R. Behavioral activation for depression: a clinician's guide. New York: Guilford Press; 2010.
9. Wallis A, Roeger L, Milan S, Walmsley C, Allison S. Behavioural activation for the treatment of rural adolescents with depression. Aust J Rural Health. 2012;20:95–6.
10. Kanter JW, Santiago-Rivera A, Santos MM, Nagy G, López M, Hurtado GD, West P. A randomized hybrid efficacy and effectiveness trial of behavioral activation for Latinos with depression. Behav Ther. 2015;46:177–92.
11. Lazzari C, Egan SJ, Rees CS. Behavioral activation treatment for depression in older adults delivered via videoconferencing: a pilot study. Cogn Behav Pract. 2011;18:555–65.
12. Chowdhary N, Anand A, Dimidjian S, Shinde S, Weobong B, Balaji M, et al. The healthy activity program lay counsellor delivered treatment for severe depression in India: systematic development and randomised evaluation. Br J Psychiatry. 2016;208:381–8.
13. World Health Organization. Mental health atlas 2011. Geneva: WHO Press; 2011.
14. Eaton J, McCay L, Semrau M, Chatterjee S, Baingana F, Araya R, et al. Scale up of services for mental health in low-income and middle-income countries. Lancet. 2011;378:1592–603.
15. Kakuma R, Minas H, van Ginneken N, Dal Poz MR, Desiraju K, Morris JE, et al. Human resources for mental health care: current situation and strategies for action. Lancet. 2011;378:1654–63.
16. World Health Organization. Mental health action plan 2013–2020. Geneva: WHO Press; 2013.
17. Andersson G, Cuijpers P. Internet-based and other computerized psychological treatments for adult depression: a meta-analysis. Cogn Behav Ther. 2009;38:196–205.

18. Andrews G, Cuijpers P, Craske MG, McEvoy P, Titov N. Computer therapy for the anxiety and depressive disorders is effective, acceptable and practical health care: a meta-analysis. Plos One. 2010;5:e13196.
19. Spek V, Cuijpers P, Nyklícek I, Riper H, Keyzer J, Pop V. Internet-based cognitive behaviour therapy for symptoms of depression and anxiety: a meta-analysis. Psychol Med. 2007;37:319–28.
20. Arjadi R, Nauta MH, Chowdhary N, Bockting CLH. A systematic review of online interventions for mental health in low and middle income countries: a neglected field. Glob Ment Health. 2015;2:1–6.
21. Asosiasi Penyedia Jasa Internet Indonesia. Profil pengguna Internet Indonesia 2014 (Internet user profile in Indonesia 2014). Jakarta: Asosiasi Penyedia Jasa Internet Indonesia; 2015.
22. Noviandari L. Hampir setengah penduduk Indonesia mengakses Internet pada tahun 2018 (Almost half of Indonesian population access Internet in 2018). 2014. https://id.techinasia.com/jumlah-pengguna-internet-indonesia-2014-2018. Accessed 10 May 2016
23. Ekers D, Richards D, McMillan D, Bland JM, Gilbody S. Behavioural activation delivered by the non-specialist: phase II randomised controlled trial. Br J Psychiatry. 2011;198:66–72.
24. Bolton P, Bass J, Neugebauer R, Verdeli H, Clougherty KF, Wickramaratne P, et al. Group interpersonal psychotherapy for depression in rural Uganda: a randomized controlled trial. JAMA. 2003;289:3117–24.
25. Patel V, Weiss HA, Chowdhary N, Naik S, Pednekar S, Chatterjee S, et al. Lay health worker led intervention for depressive and anxiety disorders in India: impact on clinical and disability outcomes over 12 months. Br J Psychiatry. 2011;199:459–66.
26. Patel V, Weiss HA, Chowdhary N, Naik S, Pednekar S, Chatterjee S, et al. Effectiveness of an intervention led by lay health counsellors for depressive and anxiety disorders in primary care in Goa, India (MANAS): a cluster randomised controlled trial. Lancet. 2010;376:2086–95.
27. Rahman A, Malik A, Sikander S, Roberts C, Creed F. Cognitive behaviour therapy-based intervention by community health workers for mothers with depression and their infants in rural Pakistan: a cluster-randomised controlled trial. Lancet. 2008;372:902–9.
28. Bockting CLH, van Valen E. Act and feel online: Internet-based behavioral activation in Dutch. Treatment module. 2015.
29. Chan A, Tetzlaff JM, Gøtzsche PC, Altman DG, Mann H, Berlin JA. SPIRIT 2013 explanation and elaboration: guidance for protocols of clinical trials. BMJ. 2013;346:e7586.
30. Kroenke K, Spitzer RL, Williams JBW. The PHQ-9: validity of a brief depression severity measure. J Gen Inter Med. 2001;16:606–13.
31. First MB, Williams JBW, Karg RS, Spitzer RL. Structured clinical interview for DSM-5—research version. Arlington: American Psychiatric Association; 2015.
32. Marks IM, Mathews AM. Brief standard self-rating for phobic patients. Behav Res Ther. 1979;17:263–7.
33. Kanter JW, Puspitasari AJ, Santos MM, Nagy GA. Behavioural activation: history, evidence and promise. Br J Psychiatry. 2012;200:361–3.
34. Rush AJ, Gullion CM, Basco MR, Jarrett RB, Trivedi MH. The inventory of depressive symptomatology (IDS): psychometric properties. Psychol Med. 1996;26:477–86.
35. Rush AJ, Giles DE, Schlesser MA, Fulton CL, Weissenburger JE, Burns CT. The inventory of depressive symptomatology (IDS): preliminary findings. Psychiatry Res. 1986;18:65–87.
36. Zimet GD, Dahlem NW, Zimet SG, Farley GK. The multidimensional scale of perceived social support. J Pers Assess. 1988;52:30–41.
37. The WHOQOL Group. Group development of the world health organization WHOQOL-BREF quality of life assessment. Psychol Med. 1998;28:551–8.
38. van Rijsbergen GD, Bockting CLH, Berking M, Koeter MWJ, Schene AH. Can a one-item mood scale do the trick? Predicting relapse over 5.5-years in recurrent depression. PLoS One. 2012;7:e46796.
39. Watson D, Clark LA, Tellegen A. Development and validation of brief measures of positive and negative affect: the PANAS scales. J Pers Soc Psychol. 1998;54:1063–70.
40. Manos RC, Kanter JW, Luo W. The behavioral activation for depression scale–short form: development and validation. Behav Ther. 2011;42:726–39.
41. Garnefski N, Kraaij V, Spinhoven P. Negative life events, cognitive emotion regulation and emotional problems. Pers Individ Dif. 2001;30:1311–27.
42. Phinney JS. The multigroup ethnic identity measure: a new scale for use with diverse groups. J Adolesc Res. 1992;7:156–76.
43. Austin MP, Frilingos M, Lumley J, Hadzi-Pavlovic D, Roncolato W, Acland S, et al. Brief antenatal cognitive behaviour therapy group intervention for the prevention of postnatal depression and anxiety: a randomised controlled trial. J Affect Disord. 2008;105:35–44.
44. Chiesa A, Mandelli L, Serretti A. Mindfulness-based cognitive therapy versus psychoeducation for patients with major depression who did not achieve remission following antidepressant treatment: a preliminary analysis. J Altern Complement Med. 2012;18:756–60.
45. Mackinnon A, Griffiths KM, Christensen H. Comparative randomised trial of online cognitive behavioural therapy and an information website for depression: 12-month outcomes. Br J Psychiatry. 2008;192:130–4.
46. Hayes AF. Beyond Baron and Kenny: statistical mediation analysis in the new millennium. Commun Monogr. 2009;76:408–20.
47. Kleinman A. Culture and depression. N Engl J Med. 2004;351:951–3.

Submit your next manuscript to BioMed Central and we will help you at every step:

- We accept pre-submission inquiries
- Our selector tool helps you to find the most relevant journal
- We provide round the clock customer support
- Convenient online submission
- Thorough peer review
- Inclusion in PubMed and all major indexing services
- Maximum visibility for your research

Submit your manuscript at www.biomedcentral.com/submit

BioMed Central
